# Revealing biases inherent in recombination protocols

**DOI:** 10.1186/1472-6750-7-77

**Published:** 2007-11-14

**Authors:** Javier F Chaparro-Riggers, Bernard LW Loo, Karen M Polizzi, Phillip R Gibbs, Xiao-Song Tang, Mark J Nelson, Andreas S Bommarius

**Affiliations:** 1 School of Chemical and Biomolecular Engineering, Parker H. Petit Institute of Bioengineering and Bioscience, School of Chemistry and Biochemistry, Georgia Institute of Technology, 315 Ferst Drive, Atlanta, GA 30332-0363, USA; 2 School of Chemistry and Biochemistry, Georgia Institute of Technology, 315 Ferst Drive, Atlanta, GA 30332-0363, USA; 3 EI DuPont de Nemours & Company, PO Box 80328, Wilmington, DE 19880-0328, USA; 4 Stheno Corporation, 311 Ferst Drive, Atlanta, 30332-0100, USA

## Abstract

**Background:**

The recombination of homologous genes is an effective protein engineering tool to evolve proteins. DNA shuffling by gene fragmentation and reassembly has dominated the literature since its first publication, but this fragmentation-based method is labor intensive. Recently, a fragmentation-free PCR based protocol has been published, termed recombination-dependent PCR, which is easy to perform. However, a detailed comparison of both methods is still missing.

**Results:**

We developed different test systems to compare and reveal biases from DNA shuffling and recombination-dependent PCR (RD-PCR), a StEP-like recombination protocol. An assay based on the reactivation of β-lactamase was developed to simulate the recombination of point mutations. Both protocols performed similarly here, with slight advantages for RD-PCR. However, clear differences in the performance of the recombination protocols were observed when applied to homologous genes of varying DNA identities. Most importantly, the recombination-dependent PCR showed a less pronounced bias of the crossovers in regions with high sequence identity. We discovered that template variations, including engineered terminal truncations, have significant influence on the position of the crossovers in the recombination-dependent PCR. In comparison, DNA shuffling can produce higher crossover numbers, while the recombination-dependent PCR frequently results in one crossover. Lastly, DNA shuffling and recombination-dependent PCR both produce counter-productive variants such as parental sequences and have chimeras that are over-represented in a library, respectively. Lastly, only RD-PCR yielded chimeras in the low homology situation of GFP/mRFP (45% DNA identity level).

**Conclusion:**

By comparing different recombination scenarios, this study expands on existing recombination knowledge and sheds new light on known biases, which should improve library-creation efforts. It could be shown that the recombination-dependent PCR is an easy to perform alternative to DNA shuffling.

## Background

Directed evolution of proteins has become a widely adopted and accepted method for protein engineering. There are two basic iterative steps involved in the process: the creation of diversity at the gene level and the screening or selection for improved variants [reviewed in [1-3]]. The quality of the diversity method is crucial and the performance of the chosen protocol has a direct impact on the success rate of obtaining improved variants as well as on the time and cost effectiveness of the ensuing screening or selection process [4,5]. Two main categories can be classified into methods for creating molecular diversity: random mutagenesis and recombination [4]. A recent, in-depth comparison of random mutagenesis methods showed that the existing methods are limited and highly biased. On average they can only achieve between 3.15–7.4 amino acid substitutions per residue [6]. On the other hand, to date recombination methods have not been compared in detail. Since its introduction in 1994, DNA shuffling of Stemmer has become a widely adopted method for creating chimeric genes. As of the end of February 2007, the two original papers outlining the methodology (one in Proceedings of the National Academy of Sciences, the other in Nature) have been cited 517 and 760 times, respectively [7,8]. DNA shuffling is the most common method with which to recombine genes, and it has become a powerful tool for protein evolution [8-23].

**Figure 1:**
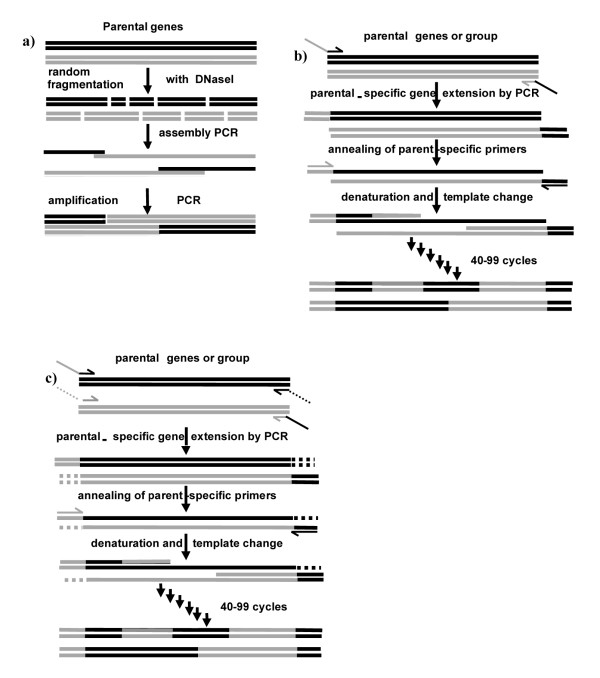
**Recombination techniques used in this work**. a) DNA Shuffling: Parental genes are randomly fragmented using DNaseI. The resulting fragments are recombined using a primer-free PCR using denaturation at high temperature, followed by annealing to other fragments, and extension by DNA polymerase. Some of these annealing events result in skew extension without recombination of fragments from two homologous parents, leading to parental background. After 35 cycles of assembly, PCR amplification with primers is used to selectively amplify full-length sequences. b) RD-PCR with one skew primer per parent: The templates are extended by parent-specific sequences resulting in asymmetric products by attaching distinct "head" and "tail". These sequences are used in the recombination PCR as primers to ensure crossover events. After the template denaturation, a high number of short annealing and extension steps results in template switching. Based on the asymmetric primers a complete product formation can only be amplified if an odd number of crossovers occurs. The resulting product will always contain different parents at the exposed ends. c) RD-PCR with two skew primers per parent: Parental templates are amplified with two unique skew primers in the first step (solid and dashed). The protocol then proceeds as in b) above, but the presence of the unique sequence prevents skew extension without recombination from happening.

Despite the pervasiveness of DNA shuffling in protein engineering, there are several drawbacks to its implementation. The protocol is somewhat skill-intensive, involving the fragmentation of the genes to be shuffled with DNAseI and a long, primerless reassembly PCR step (Figure 1). Because DNA shuffling utilizes annealing and extension steps during reassembly, crossover points are biased towards regions of high sequence identity [8,24,25]. In addition, the yield of chimeras can be quite low, particularly when short genes are being shuffled. Parental background ranging from around 20% [24,26] to almost 100% [8,27,28] has been reported. Finally, there is a lower limit to the DNA identity level of the genes being recombined, with 56% being the lowest reported identity level that lead to successful chimera generation [29].

One alternate group of methods to recombine genes are fragmentation-free PCR-based protocols, which utilize a series of short annealing/extension steps to promote template switching, which in turn, leads to recombination. The first such protocol was the **St**aggered **E**xtension **P**rocess (StEP, [30]). Further modifications that introduced skew primers to amplify chimeras over parental background have been introduced recently (**R**ecombination-**D**ependent Exponential **A**mplification-RDA-PCR [28], and **S**huffling **U**sing **U**paired **P**rim**er**s-SUUPER [31], collectively called recombination-dependent PCR, Figure 1). These recombination-dependent PCR (RD-PCR) protocols are much less skill-intensive than DNA shuffling, and the use of skew primers should, in principle, eliminate parental background.

The efficiency of diversity generation has a direct impact on the time and cost effectiveness of the screening or selection process, and ultimately, on the probability of identifying an improved variant. The optimal library generation method would be unbiased and would avoid duplication of chimeras. Reducing or completely eliminating parental background would minimize the effort required to screen these redundant variants. Additionally, the ability to control the crossover number via tunable parameters is desirable as it enables access to different areas of sequence space. It is important to note that to minimize severe disruption of chimeras the crossover region should be located in regions of similar three-dimensional structure [31].

The purpose of this work is to systematically compare the libraries produced by DNA shuffling and RD-PCR using the same representative templates, in order to determine the suitability of RD-PCR as a less labor-intensive alternative to DNA shuffling for the recombination of genes. We were interested in the number and type of chimeras generated by each protocol: the location of crossover points, the number of crossovers obtained, and the percentage of unique sequences generated with each protocol in our three test systems. We focused on RD-PCR as opposed to StEP since the use of skew primers will eliminate most parental background. Our three test cases encompass the most common scenarios encountered in protein engineering: the recombination of point mutations, recombination of closely related genes, and the recombination of low homology but structurally similar proteins (usually performed with iterative-truncation-type methods [32-34] because of the limits of DNA shuffling). To our knowledge, this is the first detailed, head-to-head comparison of DNA shuffling and RD-PCR on the same systems.

## Results and Discussion

### Recombination of point mutations using β-lactamase system

One common strategy in the directed evolution of proteins is several cycles of error-prone PCR followed by recombination of the point mutations in selected improved clones to enrich positive mutations and delete negative ones [7-13]. The optimal recombination protocol in this case would result in a high number of crossovers, no additional point mutations, and no parental background.

To estimate the crossover rate, we created a phenotype-based screening system to estimate crossover frequency on a large scale by introducing mutations into β-lactamase that disrupt activity and are not recoverable by a PCR mutation to the wild-type or a tolerated amino acid [35]. In contrast to previous systems [8,36,37], this system allows easy selection for reactivation and does not show any genetic instability that could alter the distribution between observed and actual recombination frequencies [37]. Crossovers in certain areas are required for reactivation, so an estimated crossover number can be obtained directly from the observed reactivation rate (functional complementation), reducing the need to sequence large numbers of library members. Template pairs were created requiring 1–5 crossovers for reactivation. The template pairs for DNA shuffling and RD-PCR were slightly different to allow for some extension of the genes before the first crossover in the RD-PCR pairs (see Figure S.1, additional file 1), but the nature of the point mutations and the number of crossovers required per 1000 bp was kept constant.

RD-PCR was optimized by varying DNA concentration, annealing/extension temperature, and time. A template concentration of 0.8 ng DNA/μL of PCR reaction gave sufficient yields of PCR product, while higher template concentrations reduced the crossover yield (data not shown). The results of the reactivation experiments are summarized in Table 1. Using the cycling conditions from Milano & Tang [31], the crossover rate decreases upon increasing annealing/extension time, yielding a lower survival rate on ampicillin. This is logical, as longer extension times reduce template switching, providing less opportunity for crossovers to occur. Using the cycling conditions from Ikeuchi et al. [28], increasing the annealing temperature increases the reactivation rate but decreases the yield of PCR product. Higher temperatures favor the annealing and extension of longer fragments, making it harder to begin synthesizing a recombinant gene but promoting annealing of partially extended products to different templates after the melting step.

**Table 1:** Estimation of the crossover number for β-lactamase. The number of colonies with reactivated β-lactamase (functional) is given as a percentage.

		**RD-PCR** A/E temp =						**DNA-shuffling**
		40*	45*	60^+^	60^+^	60^+^		
A/E time	CR/1000 bp	5 sec	5 sec	5 sec	15 sec	5 sec-Pfu	CR/1000 bp	< 150 bp

Min-1	1.3	59.2% ± 6.1	63.4% ± 6.6	73.3% ± 7.1	59.8% ± 6.4	66.9% ± 4.4	1.3	61.7% ± 4.3
Min-2/3	2.6	30.0% ± 4.9	33.8% ± 3.5	59.7% ± 3.8	45.6% ± 4.2	46.5% ± 2.7	2.7	38.1% ± 2.7
Min-3	4.1	19.6% ± 3.6	22.5% ± 2.9	44.0% ± 4.3	28.4% ± 3.1	35.4% ± 3.2	4.1	25.3% ± 3.7
Min-4/5	5.3	9.9% ± 2.5	13.7% ± 2.2	29.8% ± 3.3	17.6% ± 2.5	27.7% ± 2.7	5.4	16.6% ± 2.9

CR = crossovers required to gain activity

A/E = annealing/extension

*94°C for 5 min; 40 cycles of (94°C, 30 s; 40–45°C, 5 s; 72°C for 3 s); 10 cycles of (94°C, 30 s; 50°C, 30 s; and 72°C 30 s); + 94°C 2 min, 99 cycles of (94°C 1 min, 63–67°C, 5 sec), 72°C 7 min, hold at 4°C

Using *Pfu *polymerase, which has higher fidelity than *Taq *polymerase, slightly decreased the reactivation rate. However, reactivation rates were still fairly comparable to those in other conditions. In cases where avoiding the introduction of further point mutations is desirable, *Pfu *polymerase can be successfully used in RD-PCR.

DNA shuffling can be optimized either by varying the size of fragments or by adjusting the annealing temperature. Larger fragments tend to yield fewer crossovers [25,38]. Because of the high level of homology in our case, a fragment size of 50–120 bp produced sufficient product yield, though in many cases larger fragments are required to promote assembly.

In general, the reactivation rates for most RD-PCR conditions and DNA shuffling are very similar. The optimized RD-PCR conditions (60°C, 5 s) showed almost 2-fold higher crossover rates than DNA shuffling. A further advantage of RD-PCR is the ease of implementation for the RD-PCR protocol, since very low template concentrations are required (in contrast to the large amount of small DNA fragments needed for reassembly PCR) and no fragmentation is required.

### Recombination of closely related genes

Most family shuffling experiments are performed using genes from closely related organisms with DNA identity levels greater than 75%, due in part to the homology limits of the DNA shuffling protocol. To represent this scenario we chose the sequences of the red fluorescent protein from *Discosoma *sp. (DsRed, [39]) and the monomeric red fluorescent variant (mRFP, [40]). Our version of mRFP had been codon optimized for expression in *E. coli*, giving the pair a DNA identity level of 75%. The chosen template pair is still a challenging test case, since the average length of identical regions in the alignment is only 3.9 bp. The optimal result when recombining closely related genes would be a diverse library that samples all possible crossover positions. To determine crossover number, crossover position, percentage of parental background, percentage of duplicate sequences, and to estimate point mutation rate, we sequenced 295 randomly chosen functional and non-functional variants from our libraries. We also estimated the number of useful sequences for screening purposes, which is the total number of chimeras minus the number of duplicates of any sequences that appear more than once.

**Figure 2:**
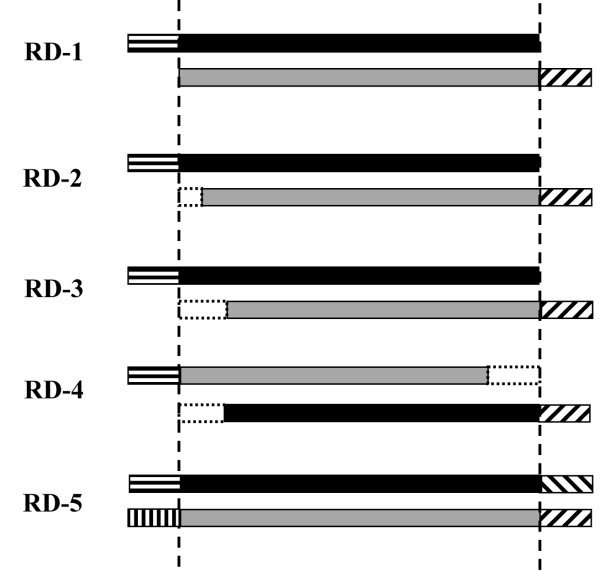
**Templates-pairs used for the recombination of mRFP (black) and DsRed (grey)**. Parental sequences or parental background are either mRFP WT or DsRed WT genes. Digested GFP pProTeT plasmids were used as cloning vectors for inserts to ensure that only fully cut plasmids were ligated with chimeric inserts.

We used a series of templates to generate RD-PCR library. Figure 2 shows the different type of templates we used for the libraries RD-PCR 1 to RD-PCR 5. For RD-PCR 1 one-sided skew templates were used. RD-PCR 2 is a combination of one sided-skew template with another parental template having a truncation near the beginning of the gene. RD-PCR 3 templates is similar to the templates used in RD-PCR 2 but with an increased truncation length. RD-PCR 4 templates are one sided-skew templates with truncations on both templates. Lastly, RD-PCR 5 uses templates that are both two sided-skewed. The effects of using different templates on the library are discussed in the next few paragraphs.

**Figure 3:**
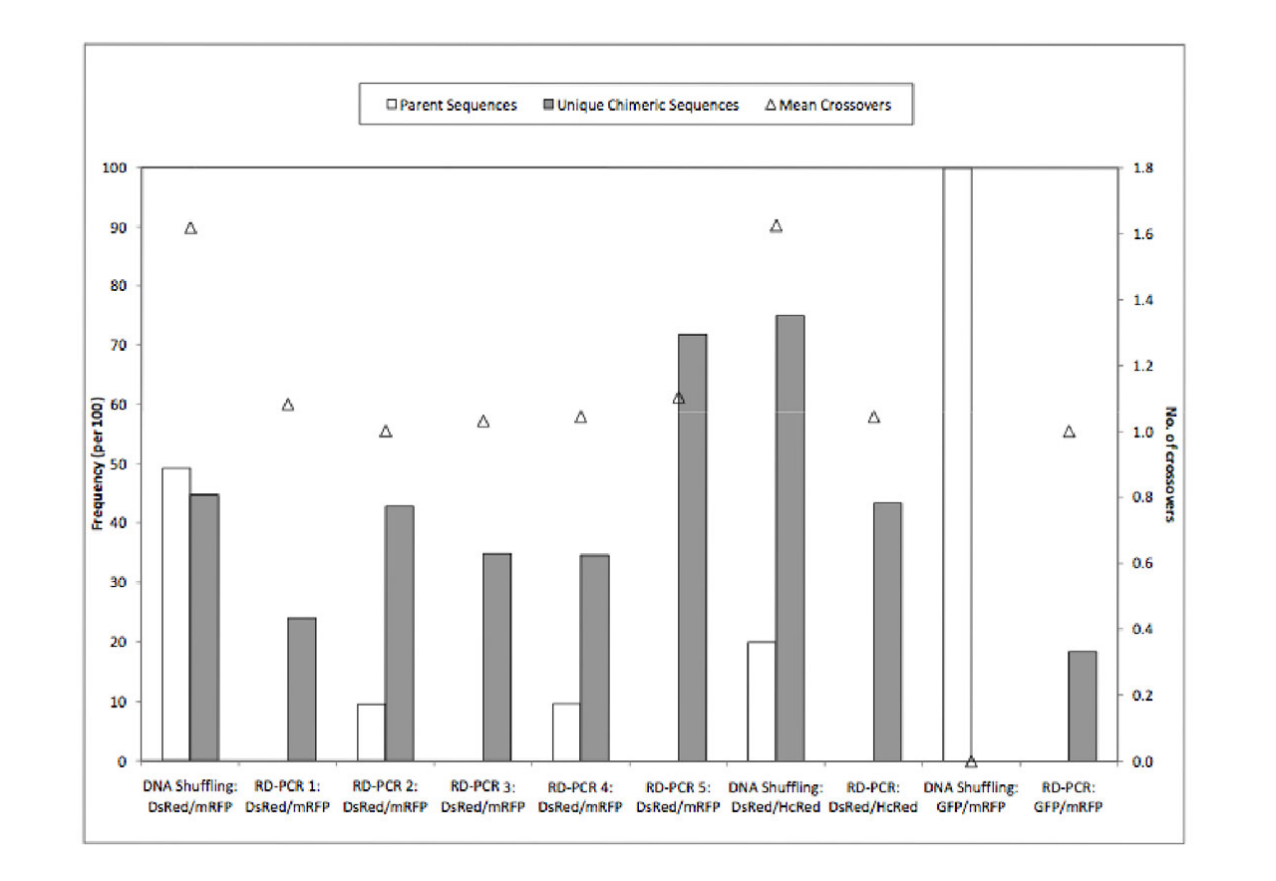
**Statistical analysis of the libraries generated by each protocol for DsRed/mRFP and GFP/mRFP**. Useful sequences are sequences that would be interesting to screen. This measure excludes parental background and does not count duplicate sequences more than once. RD-PCRs 1–4 use one skew primer per parent (Figure 1b), RD-PCR 5 uses two skew primers per parent (Figure 1c). Templates (see Figure 2): RD-PCR1 and RD-PCR 5: full length DsRed/mRFP. RD-PCR 2: DsRed template with the first 5 bp truncated, full length mRFP. RD-PCR 3: DsRed template with 44 base pairs truncated from the 5' end, full length mRFP. RD-PCR 4: Both templates are truncated. RD-PCR 5: with two skew primers per parent.

Following the procedure to recombine β-lactamase, our first RD-PCR library (RD-PCR 1) was created using a single skew primer for each parent as shown in Figure 1. We sequenced 50 variants from this library, all of which contained at least one crossover. However, 39 out of 50 contained a single crossover at position 6 (of mRFP), meaning that 38/50, or 76% were duplicate sequences, which we term chimera background. Consequently, in a screening scenario only 12 out of 50, or 24%, would be useful sequences to screen. The bias could not be removed by truncating the first 5 bases (see Figure 3) from the front of the DsRed gene before recombination (RD-PCR 2: 21 variants sequenced, 43% unique chimeras). Truncating the first 44 base pairs of the DsRed parental gene created a bias towards crossovers at the 3' end of the genes, although it was not localized to a single position (RD-PCR 3: 66 variants sequence, 35% unique chimeras). When truncating both templates simultaneously, we could only obtain clonable products when DsRed was truncated at the 3' end (43 bp) and mRFP was truncated at the 5' end (40 bp). (Note that in this case, DsRed is the "top" gene.) Fifty variants from this library (RD-PCR 4) were sequenced, and the result was a localization of crossovers to position 50 (35% unique sequences). Statistics for the libraries are shown in Figure 3.

Also striking, in the case of the truncated libraries RD-PCR 2 and RD-PCR 4, we obtained parental background of approximately 10% of sequences, despite the fact that this should not be possible when using skew primers. The parental background could arise either from contamination of the PCR reaction with full-length templates, or by the accidental elongation of the unpaired extension on a strand containing no crossovers (skew extension without recombination [41] via template switching). One way to minimize such accidental elongation would be to use two different skew primers for each parental template. Even though the recombination PCR is performed with only one primer for each parent, the amplification PCR is performed with both, thereby creating unique extensions on both ends of the gene and blocking unproductive skew extension without recombination.

**Figure 4:**
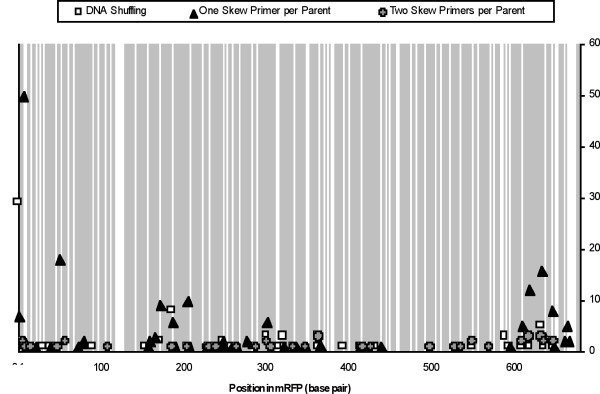
**Combined plot of the frequency and location of crossovers in libraries made from DsRed and mRFP**. Shading indicates that bases are identical in DsRed and mRFP; white space indicates that bases differ. Crossovers are denoted at the position where the first base pair differs between the two sequences. Sequences with multiple crossovers were marked at each crossover position separately. 'One skew primer per parent' combines the results from RD-PCR1-4. Subplots of Figure 4 can be found in additional file 2.

When we created the library using templates extended in both directions (RD-PCR 5), parental background was eliminated, and only chimeras were obtained. Of 39 colonies randomly sequenced, 72% contained unique sequences, predominantly with one crossover per gene. One sequence with three crossovers and one with five crossovers were obtained. Crossover points were also more evenly distributed than in the case of the libraries made with one skew primer, which showed significant bias towards the ends of the genes (Figure 4). Further details on all of the sequences obtained can be found in the supplementary information (see additional file 1).

The bias toward crossovers at the ends of PCR products amplified with single skew primers has been noted previously in recombinations during normal (as opposed to StEP-like) PCR cycling conditions [42]. By using the templates amplified with two skew primers we have demonstrated that this bias can be reduced significantly. Therefore, when performing RD-PCR the use of two skew primers for each parental template is important to avoid skew extension without recombination, which leads to parental background and a bias toward crossovers at the ends of the genes. When such precautions are taken, RD-PCR libraries result in a higher ratio of unique chimeras with lower parental background than those produced by DNA shuffling (>70% versus 45%, Figure 3). However, it is important to note that the majority of chimeras produced by RD-PCR had a single crossover (mean crossovers of 1.1), while DNA shuffling produced sequences with 2 or more crossovers (mean crossovers of 1.6), nearly 25% of the time and that the DNA shuffling parental background could also be reduced by using a skewed primer strategy similar to RD-PCR. RD-PCR is also constrained to have an odd number of crossovers (unless more than 2 parental templates are used) because the skew primers require that different parents contribute the 5' and 3' sequences. The shading on Figure 4 indicates regions of identity between mRFP and DsRed. The lines on supplementary Figures S.4 (a) to S.4 (f) represent rolling point averages of DNA identity between mRFP and DsRed. In many cases, crossovers are clustered in regions high in shading (or high DNA identity levels shown in supplementary Figures S.4 (a) to S.4 (f)) for all protocols tested. In fact, the large level of identity at the 3' end of the gene may be partially responsible for the clustering of crossover points in this region for RD-PCR using one skew primer per parent.

The optimized DNA shuffling procedure applied to mRFP and DsRed produced approximately half parental genes (67 variants, 49% background, Figure 3). The percentage of parental background is consistent with published results for the shuffling of green fluorescent protein and yellow fluorescent protein, which have a similar DNA identity level [28]. Of the 34 chimeras we obtained a mean of 1.6 crossovers. 18 had a single crossover, 11 had two crossovers, four had three crossovers, and one had four crossovers. Characteristics of the library are summarized in Figure 3 (for further details, please see Supplementary Information in additional file 1).

**Figure 5:**
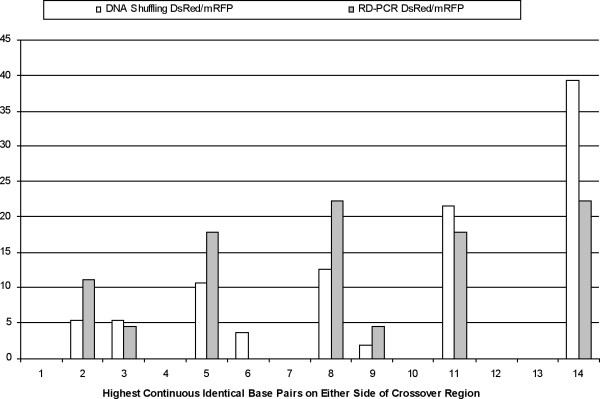
**Local identity required for a crossover to occur**. The highest number of continuous identical base pairs on either side of the crossover region is plotted versus the percentage of crossovers that contain that number. The DNA shuffling distribution differs significantly from the RD-PCR distribution as determined by the Wilcoxon Rank-Sum test (normal approximation to determine the p-value, p = 0.028).

Figure 5 shows the percentage distribution of the highest number of continuous identical base pairs on either side of the crossover region. Both protocols produced crossovers in regions with a low number of identical base pairs; however, DNA shuffling is biased towards crossovers in regions with a high level of identity (11 or more base pairs). The two distributions are significantly different as determined by the nonparametric Wilcoxon Rank-Sum test (p = 0.028). In general both protocols show a bias towards regions with a high sequence identity, as already reported for DNA shuffling [3,24,25].

It is interesting to note that we obtained more than 50% functional chimeras of DsRed and mRFP. Table S5 shows the functional relationships of some of the chimeras we obtained through recombination. A high percentage of functional chimeras should be expected as mRFP protein was evolved from DsRed protein. As a result of their high homologies, most of the crossovers preserved the activity of the parents.

### Recombination of distant homologs

In some cases it is desirable to recombine distant homologs with a low level of DNA sequence identity but a high level of structural similarity [32]. In this case, the potential for diversity increase is very large, but the probability of obtaining non-functional variants is very high. Currently, very low homology recombination is accomplished by the iterative-truncation family of methods [32-34] or by oligonucleotide-directed shuffling [43-45], because DNA shuffling cannot successfully recombine genes with very low levels of nucleotide identity below about 50%. We were interested in determining the lower limit of homology that can be successfully recombined using RD-PCR. DNA shuffling experiments were carried out simultaneously as a control measure.

**Figure 6:**
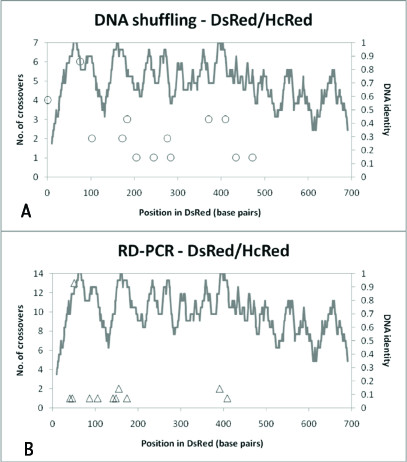
**Frequency and location of crossovers obtained from recombination of HcRed and DsRed**. **(a)**: The frequency and location of crossovers in DNA shuffled libraries obtained from HcRed and DsRed. The lines indicate rolling DNA identity calculated by summing the number of identical DNA bp in a 20 bp window and dividing by 20 bp. So, one would indicate that 100% DNA identity in a 20 bp window, ten to the left and ten to the right of a DNA residue. **(b)**: The frequency and location of crossovers in RD-PCR libraries obtained from HcRed and DsRed.

We were able to successfully recombine DsRed with HcRed (*Heteractis crispa *[46]) (65% DNA identity, near the current published lower limit for recombination using homology-based methods) with both DNA shuffling and RD-PCR. Library quality was similar to that of DsRed/mRFP – Figures 6(a) and 6(b) show that no parental background was obtained in the case of RD-PCR (23 sequences) and approximately 20% parental background was obtained for DNA shuffling (20 sequences). 56% of the crossovers for RD-PCR were localized near a 25 base pair stretch of DNA identity, whereas crossovers for DNA shuffling were more diffuse. In general, the DNA shuffling reaction appeared to yield about the same number of crossover positions, but yielded 40% more unique chimeras (14 versus 10) and many more times the clones with multiple crossovers than RD-PCR (7 of 14 different clones versus 1 of 10 different clones).

**Figure 7:**
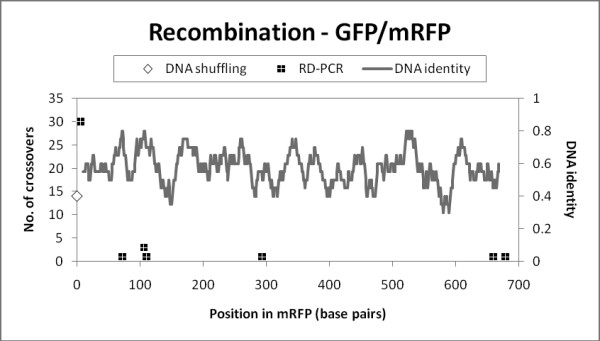
**The frequency and location of crossovers in libraries made from GFP and mRFP**. Shading indicates that bases are identical in GFP and mRFP; white space indicates that bases differ. Since the genes are of different length, gaps in the mRFP sequence were excluded.

We then moved to a lower DNA identity level, recombining GFP and mRFP (45% DNA identity). Sequencing of 38 variants from the RD-PCR showed all variants had one crossover (no parental background) and 18% useful chimeric sequences (Figure 3). Most of the sequences (30/38) contained a crossover point at the 5' end of the gene, with the remaining six unique crossover points distributed across the gene (Figure 7). Two variants had a crossover in regions with a single base pair of identity between the two sequences, highlighting the ability of PCR-based methods to produce diverse chimeras. We were unable to obtain any chimeras using DNA shuffling with this template set (14 variants sequenced, 100% parental background). We also found most of the chimeras were non functional (Table S5). In this case, the homology between GFP and mRFP could be too low for useful shuffling.

## Conclusion

To streamline the process of screening large combinatorial libraries, it is highly important to have an efficient diversity generation method, one which produces unique, nonparental sequences and is easy to implement. The current gold-standard for recombination of genes is DNA shuffling, although this protocol suffers from a high rate of parental background and can be technically difficult to perform. One recently developed alternative to DNA shuffling is RD-PCR, which is based on simple techniques and should, in theory, produce libraries with no parental background. We explored the use of RD-PCR as an alternative to DNA shuffling for three common laboratory scenarios: recombination of point mutations, closely related sequences, and distantly related homologs.

We found that RD-PCR produces libraries of equal or greater quality to DNA shuffling in the first two scenarios, as determined by the percentage of unique sequences from each protocol in the case of the fluorescent proteins and by the reactivation rate in the case of β-lactamase. Depending on the number of inactivating mutations (1 ≤ n ≤ 5), n crossovers were observed for either protocol. In the moderate homology scenario, recombination experiments for DsRed/HcRed indicate that DNA shuffling performed better than RD-PCR in producing a higher quality library with multiple crossovers In the low homology situation of GFP/mRFP (45% DNA identity level), only RD-PCR yielded chimeras.

Generally, the rate of introduction of inadvertent point mutations with RD-PCR is similar to the rate for DNA shuffling (Table 2 and [23]) performed with *Pfu *polymerase as well as for normal PCR amplifications (Table 2, all less than 5%), even though RD-PCR employs *Taq *polymerase. Even though *Taq *polymerase lacks the 5' to 3' excision-repair mechanism, RD-PCR uses a short cycling protocol. One caveat of the RD-PCR for the shuffling of fluorescence genes is the dominant finding of only one crossover per gene, while DNA shuffling resulted often in multiple crossovers. The above results imply that DNA shuffling should be the method of choice in cases where multiple crossovers are highly desired.

**Table 2:** Overall mutation rates of DNA-shuffling and RD-PCR

Source	Protocol	Overall mutagenic rate %	Total base pairs sequenced
mRFP and DsRed	RD-PCR	0.02	33900
mRFP and GFP	RD-PCR	0.04	25200
mRFP and DsRed	DNA-shuffling	0.03	21018
mRFP and GFP	DNA-shuffling	0.02	8136

DNA shuffling and RD-PCR seemed to have distinct crossover positions, hence, in some situations DNA shuffling and RD-PCR could be complementary methods used for generating diverse libraries. One can perform RD-PCR followed by DNA shuffling to improve sequence diversity of the library.

Both recombination protocols share the bias that they preferentially produce crossover in region of high sequence identity in the alignment. This phenomenon can be overcome by using homology-independent recombination protocols [33,34]. A combined approach was used by Griswold et al. [47]. They divided the genes in five sections and perform RD-PCR on four of them to obtain multiple crossovers/gene. One section showed low DNA identity (59.7% DNA identity) and they used a homology-independent recombination approach called enhanced crossover SCRATCHY [48].

To create high quality libraries with RD-PCR, two skew primers for each parental sequence must be used to minimize skew extension without recombination, such as parental background and a bias toward crossovers at the termini of the genes. If care in library design is taken, RD-PCR represents a viable alternative to classical DNA shuffling that is easier to implement. Similarly, to create high quality libraries with reduced parental background, skew primers can also be used. Such an application has been successfully tested on estrogen receptor in yeast to generate chimeras [49].

Finally, to improve success of recombination of genes with low level of identity, one can also increase sequence identity between two genes. With decreasing costs of synthesis of whole genes, designer synthetic recombination libraries can be created. It is now straightforward to resynthesize genes with new codon choices to increase DNA sequence identity between two genes prior to recombination because it is more economical to order oligonucleotides than ten years ago as the price per base-pair dropped from US $4 to approximately US $0.30 [50]. Theoretically, one can re-optimize DNA identity between two genes to prior to applying recombination to improve the chances of success and reduce bias in the library.

## Methods

### Reagents

All enzymes were purchased from New England Biolabs (Beverly, MA) except for *Pfu *polymerase, which was purchased from Stratagene (La Jolla, CA). Oligonucleotide primers were purchased from MWG Biotech (Highpoint, NC). Ampicillin, chloramphenicol and tetracycline were purchased from Sigma (St. Louis, MO). Autoclaved tetracycline was made by autoclaving 250 mg/L solution of tetracycline adjusted to pH 3 for 45 min. Mass spectrometry confirmed that approximately 60% conversion to anhydrotetracycline.

### PCR Machine

For all PCRs, we used the Eppendorf Mastercycler Gradient, Model no. 950000015 which is capable to ramping the temperature at a rate of 3.0°C/s.

### Construction of the parental plasmids

The full-length TEM-1 β-lactamase was amplified from template pDrive (Qiagen, Valencia, CA) with following primers adding a *Kpn*I and *Nde*I at the 5' and *Sal*I and *Hind*III at the 3' (restriction sites are in italic): 5'-CAA AGT TTT *G GT ACC ATA TG*A GTA TTC AAC ATT TCC GTG TCG CCC TTAT TCC C-3', 5-TAA ATA ACA *AAG CTT GTC GAC *TTA CCA ATG CTT AAT CAG TGA GGC ACC TAT CTC AGC G-3'. The PCR product was cloned into the *Kpn*I/*Hind*III site of vector pPROTet (BD Bioscience, Palo Alto, CA) resulting in pPROTet-β-lactamase.

The amino acid sequence of mRFP was obtained from NCBI and *E*. coli-codon optimized primers (Table S5) were designed using DNAworks [51] and synthesized. The mRFP gene was obtained via two PCR reactions, one to assemble the codon optimized primers, and the second to amplify the full length product. The mRFP gene was cloned using the dovetail method [52]. The gene was amplified using primers with *Esp*3I restriction sites 5'-TA*C GTC TCG *TCG ACA TGG CGT CTT CTG AAG ACG TTA TCA AAG AAT TCA TGC GT-3' and 5'-TA*C GTC TCT *GGC CTA TTA CGC ACC GGT AGA GTG ACG ACC TTC-3') and digested with Esp3I enzymes and ligated using T4 DNA ligase into *Sal*I and *Not*I digested pPROTet vector. Sequencing, expression and characterization consistent with the literature confirmed that the *E*. *coli *expression optimized mRFP gene was successfully assembled [40].

The GFP gene was amplified from pQBIT7-GFP plasmid (QBIOgene, Carlsbad, CA) using the dovetail method and primers with Esp3I restriction sites (5'-TAC GGT TA*C GTC TCG *TCG ACA TGG CGT CTT CTG AAG ACG TTA TCA-3' and 5'-TAC GGT TA*C GTC TCG *TCG ACA TGG CTA GCA AAG GAG AAG AAC TCT TCA-3'), digested using *Esp*3I and ligated into *Sal*I and *Not*I digested pPROTet vector. Expression experiments indicated successful cloning.

The DsRed gene was amplified from DsRed2-1 plasmid (BD Biosciences Clontech, Palo Alto, CA) with primers containing *Esp*3I restriction sites (5'-TAC GGT TA*C GTC TCG *TCG ACA TGG CCT CCT CCG AGA ACG TCA-3' and 5'-CAT TAC TA*C GTC TCT *GGC CTA CTA CAG GAA CAG GTG GTG GCG G-3') and cloned as with mRFP and GFP. Expression experiments indicated successful cloning.

The HcRed gene was cloned from pHcRed1-N1/1 plasmid (BD Biosciences Clontech, Plao Alto, CA) with primers containing *Sal*I and *Not*I restriction sites (CGG GAT TCC ACA TAG TCT CAG GTA *GTC GAC *ATG GTG AGC GGC CTG CTG AAG GAG AGT ATG-3' and 5'-TTC CGA TAA GTT CAT AGG CCG TG*G CGG CCG C*TC AGT TGG CCT TCT CGG GCA GGT CGC T-3'). Expression and fluorescence characterization experiments confirm that the cloning was successful.

### Introduction of point mutations into β-lactamase

The β-lactamase mutants were constructed by overlap extension PCR using the pPROTet-β-lactamase as template. Therefore following external primers were used: 5'-CCT ATC AGT GAT AGA GAT ACT GAG C-3' (top strand, N-terminal) and 5'-GAT TCT GTG GAT AAC CGT ATT ACC-3' (bottom strand C-terminal). Internal primers were used for the introduction of following mutations: K30P (AAA to CCG: 5'-CTC ACC CAG AAA CGC TGG TGC CGG TAA AAG ATG CTG AAG ATC AG-3', 5'-GAG TGG GTC TTT GCG ACC ACG GCC ATT TTC TAC GAC TTC TAG TC-3'), P105G (CCA to GGC: 5'-GAA TGA CTT GGT TGA GTA CTC AGG CGT CAC AGA AAA GCA TCT TAC G-3', 5'-CTT ACT GAA CCA ACT CAT GAG TCC GCA GTG TCT TTT CGT AGA ATG-3'), D177P (GAC to CCG: 5'-CCA TAC CAA ACG ACG AGC GTC CGA CCA ACG ATG CCT GTA GCA ATG-3', 5'-GGT ATG GTT TGC TGC TCG CAG GCT GGT TGC TAC GGA CAT CGT TAC-3'), D231P (GAT to CCG: 5'-CTT CCG GCT GGC TGG TTT ATT GCT CCG AAA TCT GGA GCC GGT GAG CGT GG-3', 5'-GAA GGC CGA CCG ACC AAA TAA CGA GGC TTT AGA CCT CGG CCA CTC GCA CC-3') and I278P (ATA to CCG: 5'-GAA CGA AAT AGA CAG ATC GCT GAG CCG GGT GCC TCA CTG ATT AAG CAT TG-3', 5'-CTT GCT TTA TCT GTC TAG CGA CTC GGC CCA CGG AGT GAC TAA TTC GTA AC-3'). The DNA sequence of each mutant was confirmed by sequencing.

### β-lactamase templates sets for the crossover determination

Four different sets of β-lactamase mutants were constructed for the estimation of the crossover number (Figure 2). The number of crossover and the segment size where the crossover must occur are summarized in Table 1. For the amplification of the templates following primers were used: Min-1: I278P (5'-CGG GAT TCC ACA TAG TCT CAG GTA GGT ACC ATA TGA GTA TTC AAC ATT TCC-3', 5'-CGA CTT ACC AAT GCT TAA TCA GTG AGG C-3'), K30P (5'-CCA TAT GAG TAT TCA ACA TTT CCG TGT CG-3', 5'-TTC CGA TAA GTT CAT AGG CCG TGG GGA TCC AAG CTT GTC GAC TTA CC-3'); Min-3a: K30P/I278P (5'-CGG GAT TCC ACA TAG TCT CAG GTA GCT TCC TTA GCT CCT GAA AAT CTC GAT AAC TC-3', 5'-CGA CTT ACC AAT GCT TAA TCA GTG AGG C-3'), D177P (5' GCT TCC TTA GCT CCT GAA AAT CTC GAT AAC TC-3', 5'-TTC CGA TAA GTT CAT AGG CCG TGG GGA TCC AAG CTT GTC GAC TTA CC-3'); Min-3b: P105G/I278P (5'-CGG GAT TCC ACA TAG TCT CAG GTA GGT ACC ATA TGA GTA TTC AAC ATT TCC-3', 5'-CGA CTT ACC AAT GCT TAA TCA GTG AGG C-3'), K30P/D177P (5'-CCA TAT GAG TAT TCA ACA TTT CCG TGT CG-3', 5'-TTC CGA TAA GTT CAT AGG CCG TGG GGA TCC AAG CTT GTC GAC TTA CC-3'); Min-5: K30P/D177P/I278P (5'-CGG GAT TCC ACA TAG TCT CAG GTA CTT TCG TCT TCA CCT CGA GTC CCT ATC AGT G-3', 5'-CGA CTT ACC AAT GCT TAA TCA GTG AGG C-3'), P105G/D231P (5'-CTT TCG TCT TCA CCT CGA GTC C-3', 5'-TTC CGA TAA GTT CAT AGG CCG TGG GGA TCC AAG CTT GTC GAC TTA CC-3').

### Template preparation for DNA shuffling

The mRFP, DsRed, HcRed, GFP and β-lactamase genes were amplified by *Pfu *polymerase using primers that anneal to the pPROTet vector (5'-CTT TCG TCT TCA CCT CGA GTC C-3', 5'-CCT ACT CAG GAG AGC GTT CAC C-3'), which added 122 bp to the 5'-terminus and 155 bp to the 3'-terminus. The PCR products were gel purified.

### DNA shuffling

DNA shuffling was performed according to Joern [24], which uses a hybrid method derived from Stemmer et al. [7] and Abècassis et al. [26]. After optimizing the DNaseI concentration and digestion time 2 μg of an equimolar mixture of the desired parental templates was digested. Fragments <120 bp (β-lactamase) or <300 bp (fluorescence proteins) were isolated by agarose gel purification using QIAEX II (Qiagen, Valencia, CA). 500–750 ng DNA-fragments were mixed with 5 μL *Pfu *buffer, 1 μL of *Pfu *polymerase and water to a final volume of 50 μl and cycled following protocol from Abècassis et al. [26]: 96°C, 90 s; 35 cycles of (94°C, 30 s; 65°C, 90 s; 62°C, 90 s; 59°C, 90 s; 53°C, 90 s; 50°C, 90 s; 47°C, 90 s; 44°C, 90 s, 41°C, 90 s; 72°C, 4 min); 72°C, 7 min; 4°C hold. Following reassembly, dilutions of the reassembled fragments were amplified using nested primers with *Pfu *polymerase and buffer to determine the optimal dilution ratio. The genes were then amplified using the optimal dilution ratio. For β-lactamase the following nested primers were used (5'-CAA AGT TTT GGT ACC ATA TGA GTA TTC AAC ATT TCC GTG TCG CCC TTA TTC CC-3', 5'-GCG ACT CTA TCC ACG GAG TGA CTA ATT CGT AAC CAT TCA GCT GTT CGA AAC AAT AAA T-3'), while for the amplification step of the fluorescent proteins the following nested primers were used (5'-ATG GGT CAT AAT CAT AAT CAT AAT CAT AAT C-3', 5'-GTC TTT CGA CTG AGC CTT TCG T-3').

### Template preparation for the recombination-dependent PCR's

For the amplification of mRFP, DsRed, HcRed, GFP and β-lactamase genes parent-specific primers were designed, which added a specific overhang (5'-CGG GAT TCC ACA TAG TCT CAG GTA-3') at the 5'-terminus of the one parent and a different overhang (5'-TTC CGA TAA GTT CAT AGG CCG TGG-3') at the 3'-terminus of the other parent (Figure 2).

### Recombination-dependent PCR

For β-lactamase, the best conditions were similar to the protocol used in SUUPER [31]: 94°C 2 min, 99 cycles of (94°C 1 min, 63–67°C, 5 s), 72°C 7 min, hold at 4°C, whereas for the fluorescent proteins, the best conditions were similar to those used by Ikeuchi [28]: 98°C for 5 min; 40 cycles of (94°C, 30 s; 40–45°C, 5 s; 72°C for 3 s); 10 cycles of (94°C, 30 s; 50°C, 30 s; and 72°C 30 s); hold at 4°C. RD-PCR reactions were performed with *Taq *polymerase unless otherwise specified.

### β-lactamase system for testing recombination of point mutations

Recombination products were digested with *Kpn*I and *Hind*III, ligated into the pPROTet vector, and transformed into *E. coli *XL1 Blue cells. We obtained around 5 × 10^5 ^transformants/μg DNA with unoptimized ligation conditions. To estimate the crossover number, the cells were plated on LB-chloramphenicol-plates (20 μg/mL). After incubating for 15 h at 37°C, plates containing between 30 and 150 colonies were replica-plated on plates containing chloramphenicol (20 μg/mL) and chloramphenicol/ampicillin (20 μg/mL/50 μg/mL), respectively. Approximately, 2500 colonies were counted in each case. The original templates were confirmed to be inactivated by streaking onto the chloramphenicol/ampicillin plates. Additionally, ten randomly picked colonies from the chloramphenicol/ampicillin plates were sequenced to confirm that reactivation was due to crossovers in the selected areas. They did not contain any of the deactivating mutations or any additional mutations.

### Sequencing of fluorescent protein variants

To analyze the fluorescent proteins, a total of 347 randomly picked variants expressing functional and non-functional proteins were sequenced to determine the crossover points, number of crossovers, and the number of continuous identical base pairs at the crossover site. We sequenced 246 RD-PCR variants and 101 DNA-shuffling variants. All sequence numbering refers to the position in mRFP.

## Authors' contributions

JCH made contribution to the conception and design of the project, the acquisition, and analysis of the data and was involved in the manuscript preparation. BWL participated in the design, acquisition and analysis of recombination of the fluorescence proteins and was involved in the manuscript preparation. KMP helped in data acquisition, the analysis of the data and in the preparation of the manuscript. PRG contributed in the conception and design of the project. XST, MJN and ASB coordinated and helped to draft the manuscript. All authors read and approved the final manuscript.

## Supplementary Material

Additional file 1 Supporting Material & Figure S.1. Contains sequencing data on all the fluorescent protein recombination experiments and a figure illustrating the various beta-lactamase variants created for recombination experiments. Click here for file

Additional file 2 Supporting Material Figures S.4 (a) to S.4 (f). Contains subplots of Figure 4. Click here for file

## References

[B1] Jaeger KE, Eggert T (2004). Enantioselective biocatalysis optimized by directed evolution. Curr Opin Biotechnol.

[B2] Bornscheuer UT, Pohl M (2001). Improved biocatalysts by directed evolution and rational protein design. Curr Opin Chem Biol.

[B3] Taylor SV, Kast P, Hilvert D (2001). Investigating and engineering enzymes by genetic selection. Angew Chem Int Ed Engl.

[B4] Lutz S, Patrick WM (2004). Novel methods for directed evolution of enzymes: quality, not quantity. Current opinion in biotechnology.

[B5] Neylon C (2004). Chemical and biochemical strategies for the randomization of protein encoding DNA sequences: library construction methods for directed evolution. Nucleic acids research.

[B6] Wong TS, Roccatano D, Zacharias M, Schwaneberg U (2006). A statistical analysis of random mutagenesis methods used for directed protein evolution. Journal of molecular biology.

[B7] Stemmer WP (1994). Rapid evolution of a protein in vitro by DNA shuffling. Nature.

[B8] Stemmer WP (1994). DNA shuffling by random fragmentation and reassembly: in vitro recombination for molecular evolution. Proceedings of the National Academy of Sciences of the United States of America.

[B9] Crameri A, Dawes G, Rodriguez E, Silver S, Stemmer WP (1997). Molecular evolution of an arsenate detoxification pathway by DNA shuffling. Nature biotechnology.

[B10] Crameri A, Whitehorn EA, Tate E, Stemmer WP (1996). Improved green fluorescent protein by molecular evolution using DNA shuffling. Nature biotechnology.

[B11] Reetz MT (2004). Controlling the enantioselectivity of enzymes by directed evolution: practical and theoretical ramifications. Proceedings of the National Academy of Sciences of the United States of America.

[B12] Zhao H, Arnold FH (1997). Functional and nonfunctional mutations distinguished by random recombination of homologous genes. Proceedings of the National Academy of Sciences of the United States of America.

[B13] Moore JC, Jin HM, Kuchner O, Arnold FH (1997). Strategies for the in vitro evolution of protein function: enzyme evolution by random recombination of improved sequences. Journal of molecular biology.

[B14] Schmidt-Dannert C, Umeno D, Arnold FH (2000). Molecular breeding of carotenoid biosynthetic pathways. Nature biotechnology.

[B15] Powell SK, Kaloss MA, Pinkstaff A, McKee R, Burimski I, Pensiero M, Otto E, Stemmer WP, Soong NW (2000). Breeding of retroviruses by DNA shuffling for improved stability and processing yields. Nature biotechnology.

[B16] Ness JE, Welch M, Giver L, Bueno M, Cherry JR, Borchert TV, Stemmer WP, Minshull J (1999). DNA shuffling of subgenomic sequences of subtilisin. Nature biotechnology.

[B17] Soong NW, Nomura L, Pekrun K, Reed M, Sheppard L, Dawes G, Stemmer WP (2000). Molecular breeding of viruses. Nature genetics.

[B18] Christians FC, Scapozza L, Crameri A, Folkers G, Stemmer WP (1999). Directed evolution of thymidine kinase for AZT phosphorylation using DNA family shuffling. Nature biotechnology.

[B19] Raillard S, Krebber A, Chen Y, Ness JE, Bermudez E, Trinidad R, Fullem R, Davis C, Welch M, Seffernick J (2001). Novel enzyme activities and functional plasticity revealed by recombining highly homologous enzymes. Chemistry & biology.

[B20] Crameri A, Raillard SA, Bermudez E, Stemmer WP (1998). DNA shuffling of a family of genes from diverse species accelerates directed evolution. Nature.

[B21] Chang CC, Chen TT, Cox BW, Dawes GN, Stemmer WP, Punnonen J, Patten PA (1999). Evolution of a cytokine using DNA family shuffling. Nature biotechnology.

[B22] Leong SR, Chang JC, Ong R, Dawes G, Stemmer WP, Punnonen J (2003). Optimized expression and specific activity of IL-12 by directed molecular evolution. Proceedings of the National Academy of Sciences of the United States of America.

[B23] Zhao H, Arnold FH (1997). Optimization of DNA shuffling for high fidelity recombination. Nucleic acids research.

[B24] Joern JM, Meinhold P, Arnold FH (2002). Analysis of shuffled gene libraries. Journal of molecular biology.

[B25] Moore GL, Maranas CD, Lutz S, Benkovic SJ (2001). Predicting crossover generation in DNA shuffling. Proceedings of the National Academy of Sciences of the United States of America.

[B26] Abecassis V, Pompon D, Truan G (2000). High efficiency family shuffling based on multi-step PCR and in vivo DNA recombination in yeast: statistical and functional analysis of a combinatorial library between human cytochrome P450 1A1 and 1A2. Nucleic acids research.

[B27] Kikuchi M, Ohnishi K, Harayama S (1999). Novel family shuffling methods for the in vitro evolution of enzymes. Gene.

[B28] Ikeuchi A, Kawarasaki Y, Shinbata T, Yamane T (2003). Chimeric gene library construction by a simple and highly versatile method using recombination-dependent exponential amplification. Biotechnology progress.

[B29] Kaper T, Brouns SJ, Geerling AC, De Vos WM, Van der Oost J (2002). DNA family shuffling of hyperthermostable beta-glycosidases. The Biochemical journal.

[B30] Zhao H, Giver L, Shao Z, Affholter JA, Arnold FH (1998). Molecular evolution by staggered extension process (StEP) in vitro recombination. Nature biotechnology.

[B31] Milano J, Tang X-S (2004). US Patent Application 20040014085.

[B32] Ostermeier M, Nixon AE, Benkovic SJ (1999). Incremental truncation as a strategy in the engineering of novel biocatalysts. Bioorganic & medicinal chemistry.

[B33] Ostermeier M, Nixon AE, Shim JH, Benkovic SJ (1999). Combinatorial protein engineering by incremental truncation. Proceedings of the National Academy of Sciences of the United States of America.

[B34] Ostermeier M, Shim JH, Benkovic SJ (1999). A combinatorial approach to hybrid enzymes independent of DNA homology. Nature biotechnology.

[B35] Huang W, Petrosino J, Hirsch M, Shenkin PS, Palzkill T (1996). Amino acid sequence determinants of beta-lactamase structure and activity. Journal of molecular biology.

[B36] Shafikhani S (2002). Factors affecting PCR-mediated recombination. Environmental microbiology.

[B37] Rozak DA, Bryan PN (2005). Offset recombinant PCR: a simple but effective method for shuffling compact heterologous domains. Nucleic acids research.

[B38] Maheshri N, Schaffer DV (2003). Computational and experimental analysis of DNA shuffling. Proceedings of the National Academy of Sciences of the United States of America.

[B39] Baird GS, Zacharias DA, Tsien RY (2000). Biochemistry, mutagenesis, and oligomerization of DsRed, a red fluorescent protein from coral. Proceedings of the National Academy of Sciences of the United States of America.

[B40] Campbell RE, Tour O, Palmer AE, Steinbach PA, Baird GS, Zacharias DA, Tsien RY (2002). A monomeric red fluorescent protein. Proceedings of the National Academy of Sciences of the United States of America.

[B41] Odelberg SJ, Weiss RB, Hata A, White R (1995). Template-switching during DNA synthesis by Thermus aquaticus DNA polymerase I. Nucleic acids research.

[B42] Meyerhans A, Vartanian JP, Wain-Hobson S (1990). DNA recombination during PCR. Nucleic acids research.

[B43] Gibbs MD, Nevalainen KM, Bergquist PL (2001). Degenerate oligonucleotide gene shuffling (DOGS): a method for enhancing the frequency of recombination with family shuffling. Gene.

[B44] Coco WM, Encell LP, Levinson WE, Crist MJ, Loomis AK, Licato LL, Arensdorf JJ, Sica N, Pienkos PT, Monticello DJ (2002). Growth factor engineering by degenerate homoduplex gene family recombination. Nature biotechnology.

[B45] Ness JE, Kim S, Gottman A, Pak R, Krebber A, Borchert TV, Govindarajan S, Mundorff EC, Minshull J (2002). Synthetic shuffling expands functional protein diversity by allowing amino acids to recombine independently. Nature biotechnology.

[B46] Gurskaya NG, Fradkov AF, Terskikh A, Matz MV, Labas YA, Martynov VI, Yanushevich YG, Lukyanov KA, Lukyanov SA (2001). GFP-like chromoproteins as a source of far-red fluorescent proteins. FEBS letters.

[B47] Griswold KE, Kawarasaki Y, Ghoneim N, Benkovic SJ, Iverson BL, Georgiou G (2005). Evolution of highly active enzymes by homology-independent recombination. Proceedings of the National Academy of Sciences of the United States of America.

[B48] Kawarasaki Y, Griswold KE, Stevenson JD, Selzer T, Benkovic SJ, Iverson BL, Georgiou G (2003). Enhanced crossover SCRATCHY: construction and high-throughput screening of a combinatorial library containing multiple non-homologous crossovers. Nucleic acids research.

[B49] Sun J, Katzenellenbogen JA, Zhao H, Katzenellenbogen BS (2003). DNA shuffling method for generating estrogen receptor alpha and beta chimeras in yeast. BioTechniques.

[B50] Voigt AC (2007). In Biology Seminar 030607, Georgia Tech, Atlanta.

[B51] Hoover DM, Lubkowski J (2002). DNAWorks: an automated method for designing oligonucleotides for PCR-based gene synthesis. Nucleic acids research.

[B52] Padgett KA, Sorge JA (1996). Creating seamless junctions independent of restriction sites in PCR cloning. Gene.

