# Supplementary material for: Revealing biases inherent in recombination protocols
Source: BMC Biotechnol. 2007 Nov 14;7:77. doi: 10.1186/1472-6750-7-77 (PMC2203992; doi:10.1186/1472-6750-7-77)
Supplement: Additional file 1 — Supporting Material & Figure S.1. Contains sequencing data on all the fluorescent protein recombination experiments and a figure illustrating the various beta-lactamase variants created for recombination experiments. [file 1472-6750-7-77-S1.doc]

# Additional File 1

**Revealing Biases Inherent in Recombination Protocols**

Javier F. Chaparro-Riggers, Bernard L.W. Loo, Karen M. Polizzi, Phillip R. Gibbs, Xiao-Song Tang, Mark J. Nelson, and Andreas S. Bommarius^*^

#### Tables

**Table S1A : Sequencing results of SUUPER on mRFP and DsRed. mRFP: 678bp, DsRed: 678bp. The crossover position refers to the numbering of mRFP.**

| Method | Genes | Crossover position | No. of crossovers | Highest Continuous bp | Frequency |
| --- | --- | --- | --- | --- | --- |
| RD-1 | mRFP, DsRed | 6 | 1 | 5 | 39 |
|  | 74.5% id | 12 | 1 | 2 | 1 |
|  |  | 78 | 1 | 5 | 1 |
|  |  | 156 | 1 | 2 | 1 |
|  |  | 159 | 1 | 5 | 1 |
|  |  | 165 | 1 | 5 | 1 |
|  |  | 186 | 1 | 14 | 1 |
|  |  | 300 | 1 | 11 | 1 |
|  |  | 414 | 1 | 8 | 1 |
|  |  | 660 | 1 | 3 | 1 |
|  |  | 6, 21, 39 | 3 | 5, 4, 8 | 1 |
|  |  | 6, 248, 300 | 3 | 5, 6, 11 | 1 |
|  |  |  |  |  | 50 sequences total |
|  |  |  |  |  |  |
| RD-2 | mRFP, DsRed | NA | 0 | NA | 2 |
|  | (5 Ft) | 6 | 1 | 5 | 9 |
|  | 74.5% | 7* | 1 | 5 | 2 |
|  |  | 72 | 1 | 5 | 1 |
|  |  | 186 | 1 | 14 | 1 |
|  |  | 204 | 1 | 14 | 1 |
|  |  | 300 | 1 | 11 | 1 |
|  |  | 417 | 1 | 8 | 1 |
|  |  | 618 | 1 | 14 | 1 |
|  |  | 633 | 1 | 14 | 2 |
|  |  |  |  |  | 21 sequences total |

| RD-3 | mRFP, DsRed | 165 | 1 | 5 | 2 |
| --- | --- | --- | --- | --- | --- |
|  | (44Ft) | 171 | 1 | 14 | 6 |
|  | 74.5% | 186 | 1 | 14 | 3 |
|  |  | 189 | 1 | 14 | 1 |
|  |  | 204 | 1 | 14 | 6 |
|  |  | 207 | 1 | 2 | 1 |
|  |  | 247 | 1 | 6 | 2 |
|  |  | 258 | 1 | 5 | 1 |
|  |  | 300 | 1 | 11 | 1 |
|  |  | 321 | 1 | 11 | 1 |
|  |  | 348 | 1 | 11 | 1 |
|  |  | 363 | 1 | 11 | 1 |
|  |  | 366 | 1 | 2 | 1 |
|  |  | 609 | 1 | 14 | 2 |
|  |  | 618 | 1 | 14 | 9 |
|  |  | 633 | 1 | 14 | 10 |
|  |  | 636 | 1 | 8 | 2 |
|  |  | 645 | 1 | 8 | 6 |
|  |  | 648 | 1 | 2 | 1 |
|  |  | 660 | 1 | 3 | 1 |
|  |  | 664 | 1 | 3 | 5 |
|  |  | 666 | 1 | 3 | 2 |
|  |  | 228, 240, 264 | 3 | 5, 8, 5 | 1 |
|  |  |  |  |  | 66 sequences total |
| RD-4 | DsRed, mRFP | NA | 1 | NA | 5 |
|  | (43Bt) (40Ft) | 50 | 1 | 5 | 18 |
|  |  | 159 | 1 | 5 | 1 |
|  |  | 171 | 1 | 14 | 3 |
|  |  | 186 | 1 | 14 | 1 |
|  |  | 190 | 1 | 14 | 1 |
|  |  | 204 | 1 | 2 | 3 |
|  |  | 231 | 1 | 8 | 1 |
|  |  | 276 | 1 | 5 | 2 |
|  |  | 300 | 1 | 11 | 1 |
|  |  | 348 | 1 | 11 | 1 |
|  |  | 438 | 1 | 5 | 1 |
|  |  | 594 | 1 | 14 | 1 |
|  |  | 609 | 1 | 14 | 3 |
|  |  | 618 | 1 | 14 | 2 |
|  |  | 633 | 1 | 14 | 4 |
|  |  | 636 | 1 | 8 | 1 |
|  | **Not possible crossover position** | 645 | 1 | 8 | 2 |
|  |  | 78, 300, 336 | 3 | 5, 11, 11 | 1 |
|  |  |  |  |  | 52 sequences total |
|  |  |  |  |  |  |
| RD-5 | DsRed, mRFP |  |  |  |  |
|  | Head & tail extension | 6 | 1 | 5 | 1 |
|  |  | 9 | 1 | 2 | 1 |
|  |  | 15 | 1 | 8 | 1 |
|  |  | 48 | 1 | 2 | 1 |
|  |  | 57 | 1 | 5 | 2 |
|  |  | 108 | 1 | 2 | 1 |
|  |  | 186 | 1 | 14 | 1 |
|  |  | 228 | 1 | 5 | 1 |
|  |  | 240 | 1 | 8 | 1 |
|  |  | 288 | 1 | 11 | 1 |
|  |  | 300 | 1 | 11 | 2 |
|  |  | 306 | 1 | 5 | 1 |
|  |  | 333 | 1 | 11 | 1 |
|  |  | 348 | 1 | 11 | 1 |
|  |  | 363 | 1 | 11 | 2 |
|  |  | 417 | 1 | 8 | 1 |
|  |  | 426 | 1 | 8 | 1 |
|  |  | 498 | 1 | 5 | 1 |
|  |  | 549 | 1 | 9 | 2 |
|  |  | 570 | 1 | 8 | 1 |
|  |  | 609 | 1 | 14 | 2 |
|  |  | 618 | 1 | 14 | 3 |
|  |  | 633 | 1 | 14 | 1 |
|  |  | **633** | 1 | **14** | 2 |
|  |  | 636 | 1 | 8 | 2 |
|  |  | 645 | 1 | 8 | 2 |
|  |  | 648 | 1 | 2 | 1 |
|  |  | 528,535,648 | 3 | 3,3,2 | 1 |
|  |  | 6,204,231,264,363 | 5 | 5,14,8,5,11 | 1 |
|  |  |  |  |  | 39 sequences total |

* - frame shift with respect to DsRed

Ft – Front (5’) truncated

Bt – Back (3’) truncated

For RD-PCR4, a sequence with a crossover at position 645 was found. In theory, this should not be a possible crossover position as the DsRed template was 3’ truncated by 43bp.

**Table S1B : Sequencing results of DNA-shuffling on mRFP and DsRed.** The crossover position refers to the numbering of mRFP.

| Method | Genes | Crossover position | No. of crossovers | Highest Continuous bp | Frequency |
| --- | --- | --- | --- | --- | --- |
| DNA-shuffling | mRFP, DsRed | NA | 0 | NA | 33 |
|  | 74.5% id | 30 | 1 | 8 | 1 |
|  |  | 39 | 1 | 8 | 1 |
|  |  | 153 | 1 | 2 | 1 |
|  |  | 171 | 1 | 14 | 1 |
|  |  | 186 | 1 | 14 | 3 |
|  |  | 247 | 1 | 6 | 1 |
|  |  | 321 | 1 | 11 | 1 |
|  |  | 417 | 1 | 8 | 1 |
|  |  | 549 | 1 | 9 | 1 |
|  |  | 589 | 1 | 3 | 1 |
|  |  | 618 | 1 | 14 | 2 |
|  |  | 633 | 1 | 14 | 3 |
|  |  | 648 | 1 | 2 | 1 |
|  |  | 90, 594 | 2 | 5,14 | 1 |
|  |  | 186, 247 | 2 | 14, 6 | 1 |
|  |  | 186, 258 | 2 | 14, 5 | 1 |
|  |  | 186, 321 | 2 | 14, 11 | 1 |
|  |  | 186, 618 | 2 | 14, 14 | 1 |
|  |  | 285, 300 | 2 | 2, 11 | 1 |
|  |  | 300, 432 | 2 | 11, 5 | 1 |
|  |  | 321,333 | 2 | 11,11 | 1 |
|  |  | 363, 589 | 2 | 11, 3 | 1 |
|  |  | 589, 594 | 2 | 3, 14 | 1 |
|  |  | 633, 645 | 2 | 14, 8 | 1 |
|  |  | 171, 393, 618 | 3 | 14, 5, 14 | 1 |
|  |  | 204, 363, 645 | 3 | 14, 11, 8 | 1 |
|  |  | 321, 609, 633 | 3 | 11, 14, 14 | 1 |
|  |  | 84, 186, 363, 414 | 4 | 5, 14, 11, 8 | 1 |
|  |  | 300, 315, 363, 636 | 4 | 11, 11, 11, 11 | 1 |
|  |  |  |  |  | 67 sequences total |

**Table S2: Sequencing results of DNA shuffling and SUUPER on DsRed and HcRed (66% sequence identity).** DsRed: 678bp, HcRed: 687 bp. Crossover position with respect to DsRed gene.

| Method | Genes | Crossover position | No. of crossovers | Highest Continuous bp | Frequency |
| --- | --- | --- | --- | --- | --- |
| RD-PCR | HcRed, DsRed | 46 | 1 | 5 | 1 |
|  | (93 Bt) (44 Ft) | 50 | 1 | 25 | 13 |
|  | 66.0% id | 86 | 1 | 15 | 1 |
|  |  | 141 | 1 | 6 | 1 |
|  |  | 148 | 1 | 6 | 1 |
|  |  | 155 | 1 | 17 | 1 |
|  |  | 174 | 1 | 17 | 1 |
|  |  | 391 | 1 | 17 | 2 |
|  |  | 409 | 1 | 17 | 1 |
|  |  | 40, 106, 155 | 3 | 6, 5, 17 | 1 |
|  |  |  |  |  |  |
|  |  |  |  |  | 23 sequences total |
| DNA-shuffling | HcRed, DsRed | NA | 0 | NA | 4 |
|  | 66.0% id | 76 | 1 | 25 | 2 |
|  |  | 102 | 1 | 15 | 1 |
|  |  | 173 | 1 | 17 | 1 |
|  |  | 184 | 1 | 9 | 1 |
|  |  | 205 | 1 | 8 | 1 |
|  |  | 275 | 1 | 17 | 1 |
|  |  | 409 | 1 | 17 | 1 |
|  |  | 102, 370 | 2 | 15, 15 | 1 |
|  |  | 275, 409 | 2 | 17, 17 | 1 |
|  |  | 283, 409 | 2 | 6, 17 | 1 |
|  |  | 370, 472 | 2 | 15, 11 | 1 |
|  |  | 76, 184 | 2 | 25, 9 | 2 |
|  |  | 76, 173, 370 | 3 | 25, 17, 15 | 1 |
|  |  | 76, 245, 433 | 3 | 25, 10, 9 | 1 |
|  |  |  |  |  | 20 sequences total |

**Table S3: Sequencing results of DNA shuffling and SUUPER on mRFP and GFP.** mRFP: 678bp, GFP: 717 bp. Crossover position with respect to mRFP gene.

| Method | Genes | Crossover position | No. of crossovers | Highest Continuous bp | Frequency |
| --- | --- | --- | --- | --- | --- |
| RD-PCR | mRFP, GFP | 12 | 1 | 5 | 30 |
|  | 45.0% id | 72 | 1 | 3 | 1 |
|  |  | 106 | 1 | 9 | 3 |
|  |  | 110 | 1 | 1 | 1 |
|  |  | 294 | 1 | 3 | 1 |
|  |  | 579 | 1 | 1 | 1 |
|  |  | 661 | 1 | 2 | 1 |
|  |  |  |  |  | 38 sequences total |
| DNA-shuffling | mRFP, GFP | NA | 0 | NA | 14 |
|  | 45.0% id |  |  |  | 14 sequences total |

**Table S4 – primers sequences used to make *E*.*coli* codon optimized mRFP gene**

| **no.** | **Primer Sequences** |
| --- | --- |
| **1** | 5'-ATGGCGTCTTCTGAAGACGTTATCAAAGAATTCATGCGTTTCAAAGT -3' |
| **2** | 5'-TCGTATGGAAGGTTCTGTTAACGGTCACGAATTCGAAATCGAAGGTG -3' |
| **3** | 5'-AAGGTGAAGGTCGTCCGTACGAAGGTACCCAGACCGCGAAACTG -3' |
| **4** | 5'-AAAGTTACCAAAGGTGGTCCGCTGCCGTTCGCGTGGGAC -3' |
| **5** | 5'-ATCCTGTCTCCGCAGTTCCAGTACGGTTCTAAAGCGTACGTTAAACACCCGG -3' |
| **6** | 5'-CGGACATCCCGGACTACCTGAAACTGTCTTTCCCGGAAGGT -3' |
| **7** | 5'-TTCAAATGGGAACGTGTTATGAACTTCGAAGACGGTGGTGTTGTTA - 3' |
| **8** | 5'-CCGTTACCCAGGACTCTTCTCTGCAGGACGGTGAATTCATCTACAA -3' |
| **9** | 5'-AGTTAAACTGCGTGGTACCAACTTCCCGTCTGACGGTCCGG -3' |
| **10** | 5'-TTATGCAGAAAAAAACCATGGGTTGGGAAGCGTCTACCGAACGTAT -3' |
| **11** | 5'-GTACCCGGAAGACGGTGCGCTGAAAGGTGAAATCAAAATGCG -3' |
| **12** | 5'-TCTGAAACTGAAAGACGGTGGTCACTACGACGCGGAAGTTAAAACC -3' |
| **13** | 5'-ACCTACATGGCGAAAAAACCGGTTCAGCTGCCGGGTGCG -3' |
| **14** | 5'-TACAAAACCGACATCAAACTGGACATCACCTCTCACAACGAAGACTACACCA -3' |
| **15** | 5'-TCGTTGAACAGTACGAACGTGCGGAAGGTCGTCACTCTACCGGTGCG -3' |
| **16** | 5'-TTACGCACCGGTAGAGTGACGACCTTC -3' |
| **17** | 5'-CGCACGTTCGTACTGTTCAACGATGGTGTAGTCTTCGTTGTGAGAGGTGAT -3' |
| **18** | 5'-GTCCAGTTTGATGTCGGTTTTGTACGCACCCGGCAGCTGAA -3' |
| **19** | 5'-CCGGTTTTTTCGCCATGTAGGTGGTTTTAACTTCCGCGTCGTAGT -3' |
| **20** | 5'-GACCACCGTCTTTCAGTTTCAGACGCATTTTGATTTCACCTTTCAG -3' |
| **21** | 5'-CGCACCGTCTTCCGGGTACATACGTTCGGTAGACGCTTCCCA -3' |
| **22** | 5'-ACCCATGGTTTTTTTCTGCATAACCGGACCGTCAGACGGG -3' |
| **23** | 5'-AAGTTGGTACCACGCAGTTTAACTTTGTAGATGAATTCACCGTCCTG -3' |
| **24** | 5'-CAGAGAAGAGTCCTGGGTAACGGTAACAACACCACCGTCTTCGAAG -3' |
| **25** | 5'-TTCATAACACGTTCCCATTTGAAACCTTCCGGGAAAGACAGTTTC -3' |
| **26** | 5'-AGGTAGTCCGGGATGTCCGCCGGGTGTTTAACGTACGCTTTAGAACCGT -3' |
| **27** | 5'-ACTGGAACTGCGGAGACAGGATGTCCCACGCGAACGGCA -3' |
| **28** | 5'-GCGGACCACCTTTGGTAACTTTCAGTTTCGCGGTCTGGGTACC -3' |
| **29** | 5'-TTCGTACGGACGACCTTCACCTTCACCTTCGATTTCGAATTCGTGA -3' |
| **30** | 5'-CCGTTAACAGAACCTTCCATACGAACTTTGAAACGCATGAATTCTTT - 3’ |

**Table S5 – Functionality of recombinants obtained from DsRed/mRFP and GFP/mRFP**

| No. | Recombination Type | No. of crossovers | Crossover postions | Fluorescent? |
| --- | --- | --- | --- | --- |
| 1 | DsRed/mRFP | 1 | 6 | Y |
| 2 | DsRed/mRFP | 1 | 12 | Y |
| 3 | DsRed/mRFP | 1 | 30 | Y |
| 4 | DsRed/mRFP | 1 | 72 | Y |
| 5 | DsRed/mRFP | 1 | 78 | Y |
| 6 | DsRed/mRFP | 1 | 156 | Y |
| 7 | DsRed/mRFP | 1 | 159 | Y |
| 8 | DsRed/mRFP | 1 | 165 | Y |
| 9 | DsRed/mRFP | 1 | 186 | Y |
| 10 | DsRed/mRFP | 1 | 204 | Y |
| 11 | DsRed/mRFP | 1 | 300 | N |
| 12 | DsRed/mRFP | 1 | 414 | Y |
| 13 | DsRed/mRFP | 1 | 417 | Y |
| 14 | DsRed/mRFP | 1 | 589 | N |
| 15 | DsRed/mRFP | 1 | 618 | N |
| 16 | DsRed/mRFP | 1 | 633 | N |
| 17 | DsRed/mRFP | 1 | 645 | N |
| 18 | DsRed/mRFP | 1 | 648 | N |
| 19 | DsRed/mRFP | 1 | 660 | Y |
| 20 | DsRed/mRFP | 1 | 664 | Y |
| 21 | DsRed/mRFP | 2 | 321, 333 | Y |
| 22 | DsRed/mRFP | 3 | 6, 21, 39 | Y |
| 23 | DsRed/mRFP | 3 | 6, 248, 300 | Y |
| 24 | GFP/mRFP | 1 | 106 | N |
| 25 | GFP/mRFP | 1 | 110 | N |
| 26 | GFP/mRFP | 1 | 293 | N |
| 27 | GFP/mRFP | 1 | 679 | N |
| 28 | GFP/mRFP | 1 | 660 | Y |

Figure S1: β-lactamase templates for the recombination of point mutations using the β-lactamase system

Since RD-PCR uses a skew primer, the amplification starts at the beginning of each template (amplification from the start codon starts with the top stand; amplification from the stop codon starts with the bottom strand), while for DNA shuffling each template can serve as a starting point. This results in differences in the minimum number of crossovers necessary for the reactivation of the β-lactamase. The minimal crossover number for the reactivation for RD-PCR is equal to 1 (Min-1), 3 (Min-2/3 and Min-3) and 5 (Min-4/5). The minimal crossover number for the reactivation for DNA-shuffling is equal to 1 (Min-1), 2 (Min-2/3), 3 (Min-3) and 4 (Min-4/5).

**Min-1**

**Min-3**

**Min-4/5**

**Min-2/3**

**45 bp**

**394 bp**

**D231P**

**P105G**

**I278P**

**D177P**

**K30P**

**742 bp**

**439 bp**

**301 bp**

**223 bp**

**214 bp**

**301 bp**

**223 bp**

**214 bp**

**162 bp**

**139 bp**

**95 bp**

**95 bp**

**209 bp**

**182 bp**

**209 bp**

**D231P**

**P105G**

**I278P**

**D177P**

**K30P**

**742 bp**

**439 bp**

**301 bp**

**223 bp**

**214 bp**

**301 bp**

**223 bp**

**214 bp**

**162 bp**

**139 bp**
