# Supplementary material for: Revealing biases inherent in recombination protocols
Source: BMC Biotechnol. 2007 Nov 14;7:77. doi: 10.1186/1472-6750-7-77 (PMC2203992; doi:10.1186/1472-6750-7-77)
Supplement: Additional file 2 — Supporting Material Figures S.4 (a) to S.4 (f). Contains subplots of Figure 4. [file 1472-6750-7-77-S2.doc]

# Additional File 2

**Revealing Biases Inherent in Recombination Protocols**

Javier F. Chaparro-Riggers, Bernard L.W. Loo, Karen M. Polizzi, Phillip R. Gibbs, Xiao-Song Tang, Mark J. Nelson, and Andreas S. Bommarius^*^

**Subplots of Figure 4:**


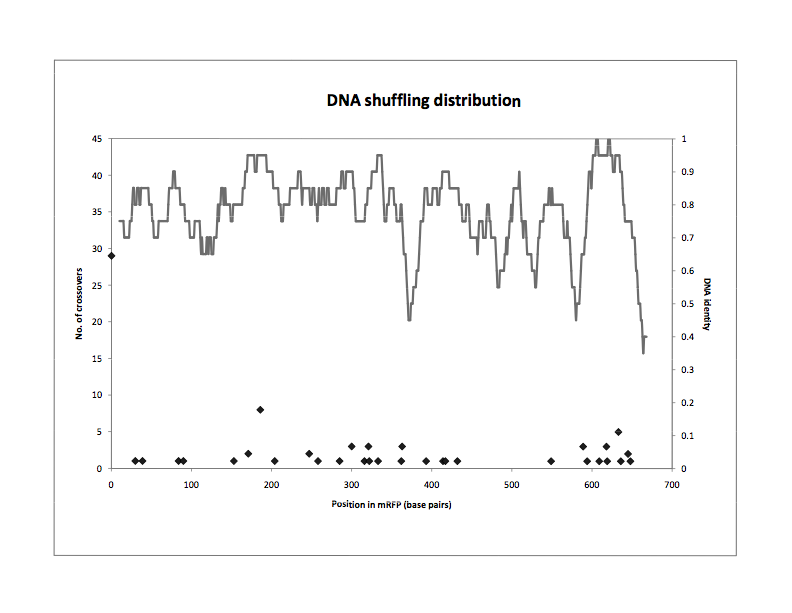


**Figure S.4 (a)**: The frequency and location of crossovers in DNA shuffling libraries made from DsRed and mRFP. The lines indicate rolling DNA identity calculated by summing the number of identical DNA bp in a 20bp window and dividing by 20 bp. So, one would indicate that 100% DNA identity in a 20bp window, ten to the left and ten to the right of a DNA residue.


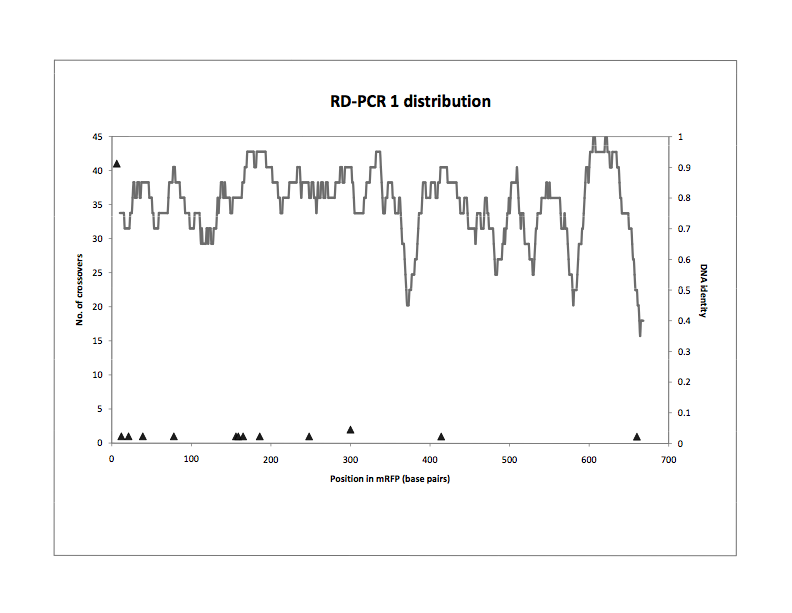


**Figure S.4 (b)**: The frequency and location of crossovers in RD-PCR 1 libraries made from DsRed and mRFP


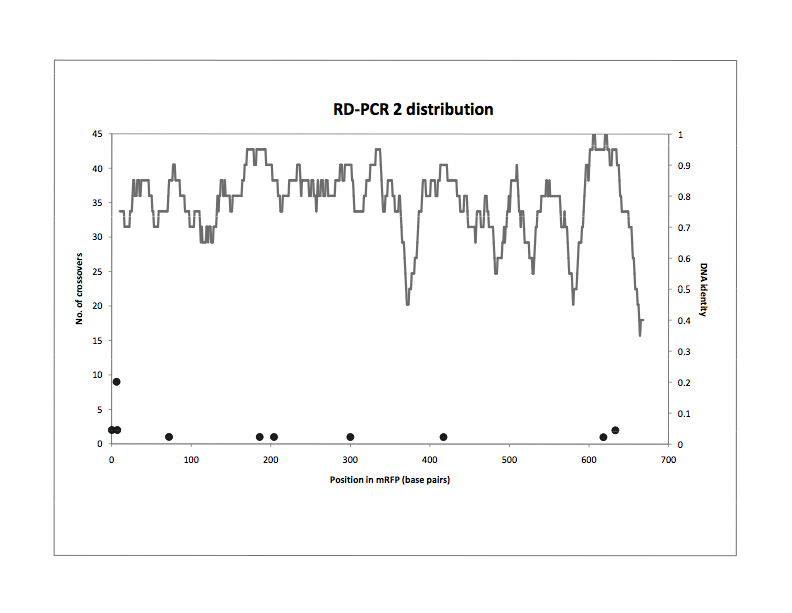


**Figure S.4 (c)**: The frequency and location of crossovers in RD-PCR 2 libraries made from DsRed and mRFP.

**
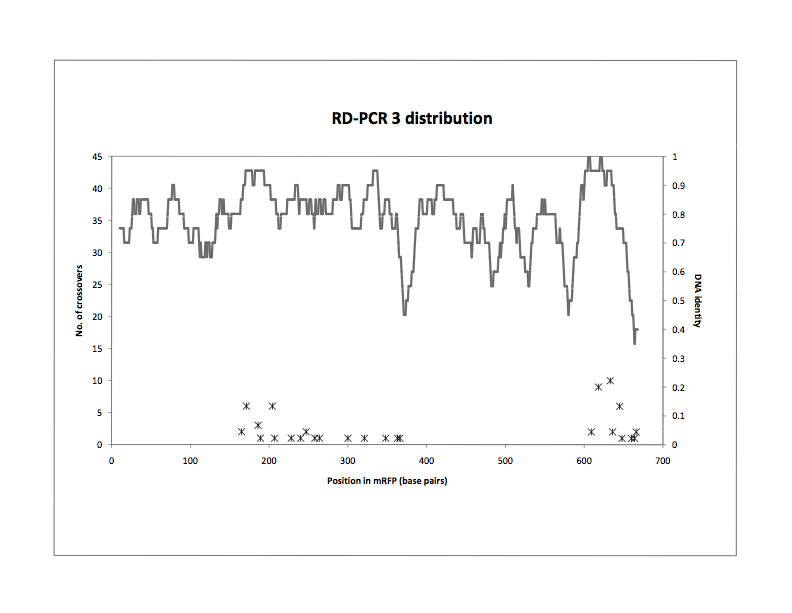
**

**Figure S.4 (d)**: The frequency and location of crossovers in RD-PCR 3 libraries made from DsRed and mRFP.


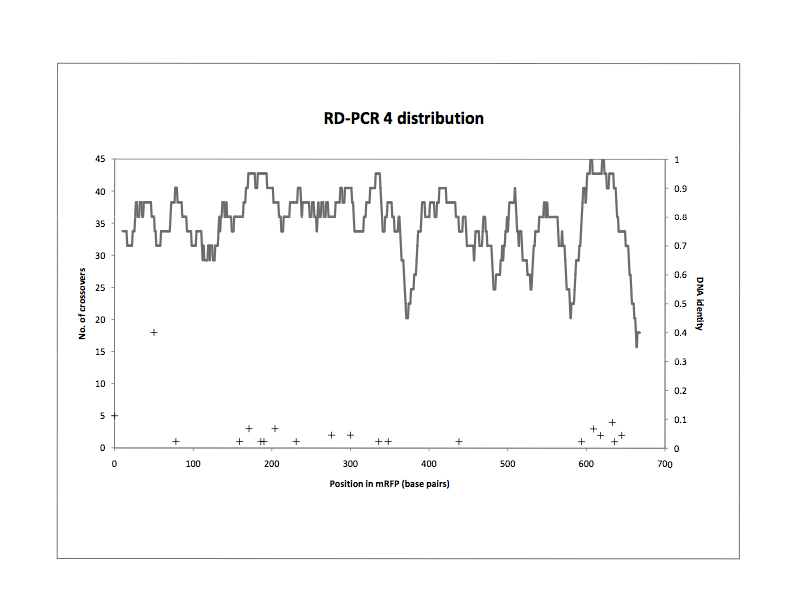


**Figure S.4 (e)**; The frequency and location of crossovers in RD-PCR 4 libraries made from DsRed and mRFP.


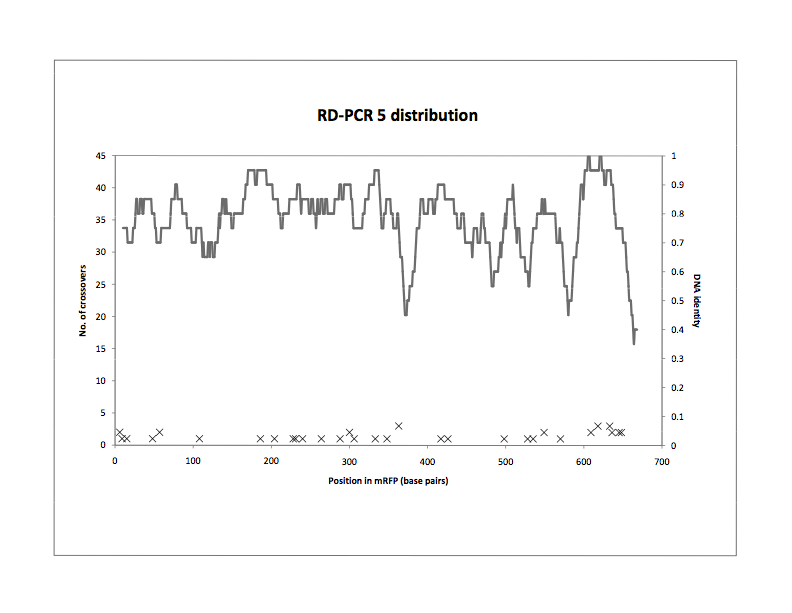


**Figure S.4 (f)**: The frequency and location of crossovers in RD-PCR 5 libraries made from DsRed and mRFP.
